# Current risk stratification and staging of multiple myeloma and related clonal plasma cell disorders

**DOI:** 10.1038/s41375-025-02654-y

**Published:** 2025-07-23

**Authors:** Saurabh Zanwar, S. Vincent Rajkumar

**Affiliations:** Division of Hematology, Mayo Clinic, Rochester, MN USA

**Keywords:** Medical research, Risk factors

## Abstract

Clonal plasma cell disorders encompass a spectrum of conditions such as multiple myeloma (MM), monoclonal gammopathy of undetermined significance (MGUS), smoldering multiple myeloma (SMM), Waldenström macroglobulinemia (WM), and immunoglobulin light chain (AL) amyloidosis. In MGUS, SMM, and MM, progression risk varies widely and is influenced by a complex interplay of tumor burden, cytogenetic abnormalities, bone marrow microenvironment, and host factors. Waldenström macroglobulinemia, while usually indolent, presents its own distinct spectrum of molecular abnormalities and disparate clinical outcomes. In AL amyloidosis, clinical trajectories are heavily dictated by the nature and extent of organ involvement. In this review, we provide a comprehensive overview of current risk stratification schema used across the spectrum of clonal plasma cell disorders, highlight the strengths and limitations of major risk stratification frameworks, and provide our recommendations for clinical practice.

## Introduction

Clonal plasma cell disorders encompass a broad and biologically diverse group of plasma cell and lymphoplasmacytic neoplasms, including multiple myeloma (MM), smoldering multiple myeloma (SMM), monoclonal gammopathy of undetermined significance (MGUS), Waldenström macroglobulinemia (WM), and immunoglobulin light chain (AL) amyloidosis. Despite sharing a common feature of clonal immunoglobulin production, these disorders differ markedly in their pathophysiology, clinical presentation, and prognosis. With the improving biologic insight into these disorders and the evolving treatment paradigms, there is an increasing need for accurate and individualized risk stratification to guide decision-making in patients with these malignancies. In this review, we examine current risk stratification approaches across the spectrum of clonal plasma cell disorders, evaluating their clinical utility, limitations, and areas for future refinement.

### Multiple myeloma

MM accounts for approximately 10% of all newly diagnosed hematologic malignancies, with over 36,000 new cases and more than 12,000 MM-related deaths annually in the United States [1]. Over the past two decades, the treatment landscape has expanded significantly, resulting in marked improvements in patient outcomes. The five-year overall survival (OS) rate has increased from 32% in the 1990s to over 60% in the past decade, and more than half of the patients without high-risk disease now have an estimated OS of more than 10 years [1, 2]. The incorporation of quadruplet induction regimens and the use of immune effector therapies in the relapsed setting are expected to further enhance these outcomes. However, a subset of patients with high-risk disease continues to experience suboptimal outcomes, with OS ranging from only 3–5 years—even with access to novel and immune-based therapies [3]. There remains a critical need for precise identification of high-risk patients and the implementation of tailored therapeutic strategies. Over the years, various prognostic models and risk stratification systems have been developed for patients with active MM. The Durie-Salmon staging system, introduced in 1975, classified patients based primarily on disease burden and represents a true staging system [4]. In 2005, the International Staging System (ISS) provided a prognostic risk-stratification system incorporating serum albumin and beta-2 microglobulin levels to classify patients into three risk categories [5]. While effective in an era of modest treatment responses, these systems were limited in capturing the underlying biologic heterogeneity of the disease. The discovery of high-risk cytogenetic abnormalities (HRCAs)—specifically del(17p), t(4;14), and t(14;16)—through interphase fluorescence in situ hybridization (FISH) prompted a shift toward cytogenetics-based risk stratification [6]. In 2014, the International Myeloma Working Group (IMWG) defined high-risk MM as International Staging System (ISS) stage II/III disease with the presence of del(17p) or t(4;14), identifying a group with a median OS of two years [7]. The Revised ISS (R-ISS) combined cytogenetic data with elevated lactate dehydrogenase (LDH) and ISS stage to define high-risk disease, which accounted for 23% of patients and had a median OS of 3.6 years [8]. The second revision (R2-ISS) further refined the prognostic weighting of cytogenetic features, incorporating 1q21 gain/amplification (1q21+) and excluding t(14;16), identifying a high-risk cohort with a median OS of 3.3 years [9]. Additionally, the Mayo Additive Staging System (MASS), a simplified model including elevated LDH and HRCAs, identified 31% of patients as high-risk, with a median OS of 4.3 years [10]. A summary of the commonly used risk stratification schema for active MM is provided in Supplementary Table 1.

Our understanding of the prognostic impact of additional genomic abnormalities continues to evolve. A markedly inferior prognosis has been noted with the presence of biallelic del(1p32) (likely due to *CDKN2C* deletions) and *TP53* mutations/inactivation [11, 12]. Similarly, there is heterogeneity in outcomes for patients with established adverse prognostic markers like 1q21+, driven in part by the associated large scale genomic events like chromothripsis/templated insertions, the presence of additional high-risk CGAs and the number of extra copies of 1q [13–15]. In patients harboring t(4;14), the location of the breakpoint of *NSD2* can determine prognosis and the presence of a ‘late-disruption’ (within the *NSD2* gene) associated with worse outcomes [16]. The *MAF* translocations, t(14;16) and t(14;20), are present in 1–3% of patients with newly diagnosed MM and enriched in APOBEC mutational signatures [17, 18]. The *MAF/MAFB* translocations are frequently associated with 1q21+ and del(17p), and it is likely the combined presence of *MAF/MAFB* translocations with secondary HRCAs that drives the adverse prognosis [19]. Majority of the patients included in the previously discussed risk stratification models were treated with what would be considered suboptimal therapy in the current era. The inclusion of a large number of variables, especially gain 1q, moved more than 50% of MM into the high-risk category, which limited the clinical meaningfulness of risk stratification.

**Fig. 1:**
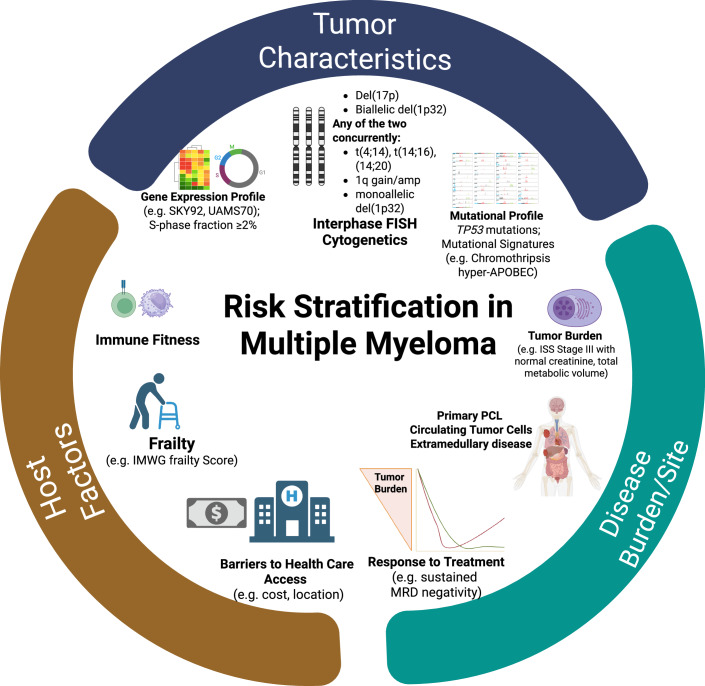
Risk stratification in multiple myeloma. An evolving paradigm of risk stratification for multiple myeloma involves comprehensive tumor genomics evaluation, consideration for disease burden and location, and various host factors that can determine access and response to existing treatments (Created using Biorender^®^). ISS international staging system, IMWG International Myeloma Working Group, FISH fluorescence in situ hybridization, MRD measurable residual disease, PCL plasma cell leukemia.

In response, the International Myeloma Society (IMS) and IMWG have introduced a new risk stratification framework in 2024 (Table 1) [20]. This classification defines high-risk MM as the presence of del(17p) and/or *TP53* mutation and biallelic del(1p32) by themselves. The presence of an elevated beta-2 microglobulin (>5.5 mg/L) in the setting of a normal serum creatinine (<1.2 mg/dL) is associated with inferior survival and an independent high-risk feature in the new IMS/IMWG classification [20]. Additionally, patients harboring two or more intermediate-risk abnormalities—1q21+ (gain or amplification), t(4;14), t(14;16), monoallelic del(1p32)—are also classified as high-risk [20]. Using this new schema, approximately 20% of patients were identified as high-risk. We recommend adopting this new IMS/IMWG definition for consistent and robust risk stratification in newly diagnosed multiple myeloma. Using a uniform risk-stratification schema will allow for harmonization of efforts in conducting clinical trials focused on high-risk MM. As we move into an era of risk-adapted therapy, further refinement to these schemas is expected by incorporation of tumor-specific factors like presence of circulating tumor cells and extramedullary disease, high-risk gene expression profiles, host specific factors like frailty and immune fitness, and response parameters like measurable residual disease (Fig. 1) [21–30].

**Table 1:** The International Myeloma Society (IMS)/International Myeloma Working Group (IMWG) Definition for High-risk Multiple Myeloma.

Presence of any one of the following:
• Del(17p)^a^ and/or *TP53* mutation^b^ • Biallelic del(1p32) • t(4;14), t(14;16) or t(14;20) Co-Occuring with 1q21+ ^c^ or monoallelic del(1p32) • Monoallelic del(1p32) Co-occuring with 1q21+^c^ • Elevated beta-2 microglobulin (>5.5 mg/dL) with normal renal function (creatinine <1.2 mg/dL)
Mayo Supplemental Criteria for High-Risk Multiple Myeloma
• Primary plasma cell leukemia • Newly diagnosed myeloma with extramedullary disease • High Plasma Cell S-phase Fraction (≥2%)
Mayo Supplemental Criteria for Double-Hit Multiple Myeloma
• Two or more of the 4 IMS/IMWG high risk qualifying abnormalities listed above with the exception of elevated beta-2 microglobulin

^a^At least a 20% cancer clonal fraction in CD138-sorted plasma cells.

^b^Assessed by a next generation dequencing-based method.

^c^gain (3 copies) or amplification (4 or more copies) of chromosome 1q.

### Monoclonal gammopathy of undetermined significance

Multiple myeloma consistently develops from the premalignant precursor condition, termed MGUS [31]. MGUS is defined by the presence of a circulating monoclonal (M) protein <3 g/dL, clonal bone marrow plasma cells (BMPCs) < 10%, and no evidence of end-organ damage attributable to the plasma cell disorder [32]. While MGUS is relatively common in the general population, affecting 3–6% of adults over the age of 50, only a small proportion of individuals with MGUS will ever develop clinical consequences [33, 34]. The annual risk of progression from MGUS to MM or related malignancies requiring treatment is approximately 1% per year [35]. However, there is substantial heterogeneity in progression risk among patients with MGUS. In a Mayo Clinic study of 1384 patients with MGUS, the presence of three of the following risk factors—an abnormal serum free light chain (sFLC) ratio, an M-protein concentration ≥1.5 g/dL, or a non-IgG isotype—was associated with a 58% risk of progression to MM over 20 years [36]. In contrast, patients without any of these risk features had only a 5% risk of progression over the same period. This risk stratification model, subsequently adopted by the IMWG, is recommended for evaluating patients with MGUS at initial diagnosis (Table 2) [37]. Notably, longitudinal application of the IMWG model at follow-up continues to demonstrate robust prognostic value [38]. Presence of immunoparesis (characterized by suppression of 2 or more uninvolved immunoglobulins) and a high fraction of clonal plasma cells in the bone marrow are other important risk factors associated with an early progression of MGUS [39–41].

**Table 2:** Risk stratification of monoclonal gammopathy of undetermined significance (MGUS).

IMWG Risk Category	Relative Risk of Progression	Absolute Risk of Progression at 20 years (%)	Absolute Risk of Progression at 20 years, accounting for death as a competing risk (%)
Low risk • M-protein <1.5 g/dL • normal sFLCR (0.26-1.65) • IgG isotype	1	5	2
Low-intermediate risk (any 1 factor being abnormal)	5.4	21	10
High-Intermediate Risk (any 2 factors abnormal)	10.1	37	18
High-risk (all three factors abnormal)	20.8	58	27

*IMWG* international myeloma working group, *sFLCR* serum free light chain ratio [^36^]. © the American Society of Hematology.

Risk stratification in MGUS plays a critical role in guiding baseline evaluation and surveillance strategies. For example, in low-risk MGUS, the probability of identifying ≥10% BMPCs or skeletal lesions at baseline is typically <5%, making extensive diagnostic workup potentially unnecessary [42]. Similarly, after demonstrating clinical stability over the first 6–12 months, follow-up for low-risk MGUS can be performed at less frequent intervals. The iStopMM study has further refined the predictive capacity of the IMWG model by incorporating serum immunoglobulin levels alongside the original three variables to better predict BMPC infiltration [43].

### Smoldering multiple myeloma

SMM is defined by the presence of ≥3 g/dL of serum monoclonal protein and/or at least 10% clonal BMPCs with the absence of myeloma defining events [31]. Unlike MGUS, the risk of progression of SMM to active MM is significantly higher at around 10% per year for the first 5 years after diagnosis [44]. Till recently, the exact prevalence of SMM was unclear as routine bone marrow aspirates were not performed for patients with MGUS. The previous estimates of 0.44 cases per 100,000 individuals from the Swedish Cancer Registry likely underestimate its prevalence, given this was lower than the estimated MM rate [41]. The iStopMM study screened 75,422 individuals (51% of all individuals above the age of 40 in Iceland) and identified SMM in 0.53% of the eligible screened population [45].

Within SMM, it is critical to identify patients with a high risk of progression who can benefit from early intervention and enrollment in clinical trials. Various risk stratification models have been utilized to determine the risk of progression in SMM. The Spanish model, using ≥95% aberrant plasma cell phenotype on flow cytometry and reduction in uninvolved immunoglobulin (score of 1 for each), identified a cohort of high-risk SMM (score of 2) with a median time-to-progression (TTP) of 23 months [46]. Subsequently, the Mayo 20/2/20 risk-stratification including 421 patients with SMM that were stratified into three risk groups based on their BMPCs (>20%), serum M-protein (>2 g/dL), and sFLC ratio (>20), Table 3 [47]. The median TTP for high-risk SMM, as defined by the 20/2/20 criteria, was 29 months. This was validated in a large international cohort, which reported a 2-year progression risk of 44% for patients with 2 or more risk factors based on the Mayo 20/2/20 criteria [48]. Biologically most patients with low and intermediate risk SMM, and almost all patients with high-risk SMM have malignancy signatures identical to that seen in MM.

**Table 3:** The Mayo 20/2/20 risk stratification schema for smoldering multiple myeloma.

Risk Category	Time-to-Progression (median, months)	Estimated Rate of Progression at 2 years from Diagnosis, %	Relative Risk of Progression at 2 years from Diagnosis
Low Risk None of the following: BMPCs >20% M spike >2 g/dL sFLC ratio >20	110	9.7	1 (reference)
Intermediate Risk: Presence of 1 of the three risk factors	68	26.3	2.71
High-risk: presence of two or three risk factors	29	47.4	4.89

Adapted from ref. [^47^].

Further refinement of the Mayo 20/2/20 risk-stratification model can be accomplished if cytogenetic results are available. The IMWG assessed the impact of FISH data, with t(4;14), t(14;16), +1q, and del(13q) noted to be associated with a shorter TTP. Incorporating this FISH data into the Mayo model, a subset of patients with three or more risk factors demonstrated a 2-year progression risk of 63% [48]. The application of the 20/2/20 model at subsequent time points in follow-up has also demonstrated clinical utility [49]. Similarly, evolving patterns of serum monoclonal protein during follow-up evaluations may further enhance risk stratification in SMM [50]. As we move closer to exploring treatment strategies for high-risk SMM, having a robust risk-stratification tool is essential [51]. We recommend the 20/2/20 risk stratification schema as a reliable framework for assessing progression risk in SMM given its simplicity and reproducibility.

### Waldenström macroglobulinemia

WM is an IgM-secreting lymphoplasmacytic lymphoma (LPL) that arises from activated B-cells [52]. Each year, ~1500 individuals are diagnosed with WM in the United States, with an age-adjusted incidence rate of 0.42 per 100,000 person-years [53]. About 20% of patients have a preceding diagnosis of smoldering WM (SWM) before progressing to active or symptomatic disease [54]. Smoldering WM is defined by ≥10% bone marrow infiltration by LPL and/or an M-protein level ≥3 g/dL in the absence of WM-related symptoms requiring treatment. These symptoms include hemoglobin ≤10 g/dL, platelet count ≤100 × 10^9^/L, significant fatigue, B symptoms, symptomatic organomegaly, hyperviscosity, cryoglobulinemia, histologic transformation, ALH amyloidosis, renal insufficiency, and clinically significant sensorimotor peripheral neuropathy attributable to WM [55, 56]. Multiple risk factors and risk stratification systems have been developed over the years in WM. In a cohort of 489 patients with asymptomatic WM and IgM MGUS, the following were identified as independent risk factors for progression: serum IgM ≥4500 mg/dL, bone marrow LPL infiltration ≥70%, serum beta-2 microglobulin ≥4 mg/dL, and serum albumin <3.5 g/dL [57]. These variables were incorporated as continuous factors in a proportional hazards model and stratified into quartiles. The median time to progression (TTP) was 1.8 years for the high-risk group (above the third quartile), 4.8 years for the intermediate-risk group (interquartile range), and 9.3 years for the low-risk group (below the first quartile) [57]. Notably, this model did not incorporate *MYD88*^*L265P*^ and *CXCR4* mutation status due to insufficient data available at that time. The *MYD88*^*WT*^ genotype is associated with a significantly shorter TTP to active WM (median TTP: 1.8 years; hazard ratio: 2.7) [57]. Similarly, patients with *CXCR4* mutations experienced a shorter TTP (median 51 months vs. not reached in *CXCR4*^*WT*^) [58]. Importantly, patients with SWM have an overall survival (OS) comparable to the general population, and even those with high-risk SWM should not initiate therapy unless treatment criteria are met [54].

Among patients with active WM, the median OS approaches a decade, although outcomes are heterogeneous [59]. The International Prognostic Scoring System for WM (IPSS-WM) stratifies patients into three risk categories based on five factors: age, platelet count, hemoglobin level, beta-2 microglobulin, and IgM level. The high-risk group demonstrated a 5-year OS of 36% [60]. However, the IPSS-WM was developed in a cohort of patients treated before 2001 and did not interrogate the prognostic impact of the *MYD88*^*L265P*^ mutation, which was discovered subsequently in WM. More recently, the Modified Staging System for WM (MSS-WM) has been proposed, stratifying patients into four risk groups based on age, serum LDH, and serum albumin. In the derivation cohort, the 5-year OS ranged from 55% in the highest-risk group to 93% in the lowest (Table 4). These findings were replicated in an external validation cohort, wherein the 5-year OS was 57% for the highest risk group and 93% for the lowest risk group. The MSS-WM maintained prognostic significance even when accounting for non-WM-related mortality as a competing risk and performed better than the IPSS-WM [61]. The *MYD88*^*L265P*^ was not identified to be prognostic in the derivation or validation cohorts in MSS-WM [61]. Incorporating elevated beta-2 microglobulin (≥4 mg/dL) into high-risk MSS-WM may identify an ultra-high risk cohort with a median OS of 2.5 years [61]. Given its simplicity, external validation, and relevance in the modern treatment landscape, we recommend using the MSS-WM for risk stratification in active WM. It is worth noting that most patients in the MSS-WM cohorts received frontline chemoimmunotherapy, the current standard of care. Future efforts should aim to validate the MSS-WM in cohorts treated with frontline BTK inhibitors and evaluate the prognostic utility of *CXCR4* mutational status.

**Table 4:** Risk Stratification for Active/Symptomatic WM using the Modified Staging System for Waldenström Macroglobulinemia (MSS-WM).

Risk Category	Proportion of patients in each stratum, %	Estimated median overall survival (OS), years	5-year OS in the derivation cohort, %
Score Assignment− Serum albumin <3.5 g/dL: 1 point Age 66–75 years: 1 point Age >75 years: 2 points Serum LDH > ULN: 2 points			
Low Risk (Score 0)	21	14.6	93
Low-Intermediate Risk (Score 1)	32	11.2	82
Intermediate Risk (Score 2)	24	8.3	69
High Risk (Score ≥3)	23	5.5	55

Adapted from ref. [^61^].

*LDH* lactate dehydrogenase, *ULN* upper limit of normal.

### AL Amyloidosis

AL amyloidosis is characterized by the excess production of immunoglobulin light chains (or rarely heavy chains) secondary to a clonal plasma cell disorder, that are deposited as misfolded proteins in various organs resulting in the consequent damage [62]. In a Mayo Clinic study analyzing individuals residing in Olmsted County between 1900 and 2015, the incidence of AL amyloidosis was found to be 1.2 per 100,000 person-years, with an estimated 2200–3500 new cases diagnosed annually in the United States [63, 64]. Globally, the incidence is estimated at 3 to 12 cases per million person-years [65].

Prognosis in AL amyloidosis is heavily influenced by the extent of organ involvement, with cardiac involvement consistently associated with poorer outcomes. Multiple staging systems have been developed over time to help stratify risk (Table 5). The Mayo Clinic 2004 staging system, based on troponin T (>0.035 ng/mL) and NT-proBNP (>332 pg/mL), stratified patients into three risk groups, and those in the high-risk category had a median overall survival (OS) of 3.5 to 4 months [66]. In 2013, a European modification of the Mayo 2004 system identified an ultra-high-risk cohort (Stage IIIB), defined by troponin *T* > 0.035 ng/mL and NT-proBNP >8500 pg/mL or systolic blood pressure <100 mm Hg. This group had a median OS of 3 months, with a hazard ratio (HR) for mortality of 11.1 compared to Stage I patients with normal cardiac biomarkers [67]. The Mayo Clinic 2012 staging system incorporated the difference in free light chains as an additional prognostic marker, alongside the established cardiac biomarkers. This model stratified patients into four risk groups with more balanced distribution. The median OS for Stage IV patients was 5.8 months, with a hazard ratio for death of 6.3 compared to Stage I [68]. Comparatively, the Mayo 2012 system provides better prediction of long-term survival, while the Mayo 2004 system with the European modification offers more accurate identification of patients at risk of early mortality [62, 69]. For this reason, we recommend using both the Mayo 2012 model and the modified Mayo 2004 model for baseline risk assessment. With the advent of high-sensitivity troponin-T, revised cutoffs have been proposed and validated. Conversions for these are described elsewhere [70]. A BNP and cardiac troponin-I-based risk stratification model has also been proposed by the Boston University investigators and demonstrates excellent concordance with the Mayo Clinic 2004 model [71]. In patients with renal AL amyloidosis, one of the important clinical relevant endpoints is the risk of dialysis dependence. Palladini et al. demonstrated that estimated glomerular filtration rate (eGFR) of <50 ml/min/1.73 m^2^ and 24 h proteinuria >5 g/dL reliably predicted the risk of dialysis dependence, with a 3-year rate of 60–85% when both these risk factors were present [72]. With advances in treatment options for AL amyloidosis and incorporation of daratumumab in the frontline treatment, early mortality has started to decrease, and the relevance of these prognostic markers in the current treatment era remains to be investigated [73, 74].

**Table 5:** Risk Stratification Models and Staging Systems for AL Amyloidosis.

Risk Stratification and Variables	Stages and proportion of patients	HR and Median OS for Mortality (95% CI)
Mayo Clinic 2004 Risk Stratification with European Modification • Troponin T ≥ 0.035 ng/mL (1 point) • NT-proBNP ≥332 pg/mL (1 point) • NT-proBNP >8500 pg/mL for Stage IIIB	Stage I (Score 0): Both Troponin T and NT-proBNP below threshold Stage II (Score 1): One of two markers above threshold Stage III (Score 2): both markers above cutoff European Modification (Stage IIIB): Troponin T ≥ 0.035 ng/mL and NT-proBNP>8500 pg/mL	Stage I: HR: 1.0 (reference) Median OS: 26.4 months Stage II: HR: 2.5 Median OS: 10.5 months Stage III: HR: 6.7 Median OS: 3.5 months [Stage IIIA- NT-proBNP ≤8500 pg/mL: HR of 4.9] Stage IIIB: HR: 11.1
Mayo Clinic 2012 Model • dFLC≥18 mg/dL (1 point) • cTnT ≥ 0.025 ng/mL (1 point) • NT-proBNP ≥ 1800 pg/mL (1 point)	Stage I (Score 0): None of the marker above cutoff Stage II (Score 1): One of the three markers above cutoff Stage III (Score 2): any two markers above cutoff Stage IV (Score 3): All three markers above cutoff	Stage I: HR:1.0 (reference) Median OS: 94.1 months [5-year OS: 59%] Stage II: HR: 1.7 Median OS: 40.3 months [5-year OS: 42%] Stage III: HR: 4.1 Median OS: 14 months [5-year OS: 20%] Stage IV: HR: 6.3 Median OS: 5.8 months[5-year OS: 14%]
Renal AL staging	Stage	3-year risk of dialysis dependence
Risk Factors: • eGFR >50 ml/min/1.73 m^2^ (normalized body surface area) • 24 h proteinuria >5 g	Stage I: neither risk factor present Stage II: one of the two risk factors present Stage III: both risk factors present	0% in the derivation cohort 4% in the validation cohort 15% in the derivation cohort 30% in the validation cohort 60% in the derivation cohort 85% in the validation cohort

*cTnT* cardiac troponin T, *dFLC* difference in involved and uninvolved serum free light chains, *eGFR* estimate glomerular filtration rate, *HR* Hazard Ratio, *OS* overall survival.

### Summary

Risk stratification in plasma cell disorders is inherently complex, requiring a nuanced assessment that integrates clone-related characteristics, tumor burden, and host-specific factors. As we advance into an era of increasingly personalized therapy, accurately identifying patients at highest risk of mortality becomes essential—not only to tailor therapeutic interventions but also to prioritize the development of novel treatment strategies aimed at improving outcomes. Conversely, individuals projected to have excellent long-term survival may benefit from approaches that reduce treatment intensity. For these patients, exploring de-escalation strategies holds the promise of minimizing toxicity and preserving quality of life without compromising efficacy. At the core of these advances lies accurate and personalized risk assessment, which serves as the foundation for truly individualized care. In this review, we have summarized the current state-of-the-art risk stratification systems that we recommend for clinical use when treating patients with the spectrum of clonal plasma cell disorders.

## Supplementary information

Supplementary Table 1
