# Supplementary material for: Current risk stratification and staging of multiple myeloma and related clonal plasma cell disorders
Source: Leukemia. 2025 Jul 23;39(11):2610–7. doi: 10.1038/s41375-025-02654-y (PMC12589131; doi:10.1038/s41375-025-02654-y)
Supplement: Supplementary file 1 — Supplementary Table 1 [file 41375_2025_2654_MOESM1_ESM.docx]

| Supplementary Table 1. Risk Stratification Models in Newly Diagnosed Multiple Myeloma | | | | | | |
| --- | --- | --- | --- | --- | --- | --- |
| Model (n=derivation cohort), year | Clinical Parameters | High-Risk Cytogenetic Abnormality (HRCA) Definition | Definition of HRMM | Risk Groups and Proportion clssified as HRMM | PFS/OS for HRD (months) | Strenghts and Limitations |
| IMWG 2014 Classification^7^  (n=2642) | ISS Stage (albumin, β2M) | t(4;14),  del17p | ISS II/III + HRCA | 3 strata;  HRMM:20% | 4-yr PFS/OS:  11%/33%;  Median OS 2 years | Early models incorporating cytogenetic data for prognostication;  Developed in the era of older treatment strategies; limited cytogenetic data input for high-risk designation |
| R-ISS^8^  (n=3060), 2015 | ISS Stage (albumin, β2M), LDH | t(4;14),  del17p,  t(14;16) | ISS III + LDH > ULN OR HRCA | 3 strata;  HRMM10% | 5-yr PFS/OS:  24%/40%  Median: 29m/43m |  |
| Myeloma Genome Project^11^  (n=1273), 2019 | ISS III | WES based:  Biallelic TP53 inactivation OR  Amp (≥4 copies) 1q | Biallelic TP53 loss  OR  ISS III AND 1q amp | 3 strata;  Double hit grup: 6.1% | Median PFS/OS:  15.4m/20.7m | Large cohort of patients with WES data;  Small cohort with ultra- high-risk designation |
| IFM Cytogenetic Prognostic Index^12^, 2019 (n=647 for training set) | None | t(4;14)=0.4 pts; del(17p)=1.2 pts; 1q gain=0.5 pts; del1(p32)=0.8 pts; trisomy 5= 0.3 pts; trisomy 21=0.3 pts | Score >1 | 3 strata;  HRMM 18% | Median OS:  26-34 months | Developed on patients included in clinical trials; Relatively small cohort;  No clinical parameters incorporated. |
| R2-ISS^9^  (n=2226 for training set), 2022 | ISS Stage:  II (1 pt)  III (1.5 pts)  LDH>ULN (1 pt) | Del17p (1.5 pts)  t(4;14): 1 pt  1q+ (0.5 pts) | Composite Score of 3-5 | 4 strata;  HRMM 9 % | 5-year PFS/OS:  17%/37%  Median:  19.9m/37.9m | Clinical Trial Cohort;  Small cohort with high-risk designation; high proportion of missing data. |
| Mayo Additive Scoring System^10^  (n=1327 for training set), 2022 | ISS Stage III  (1 pt)  LDH>ULN (1 pt) | Del17p (1pt)  t(4;14): 1pt  t(14;16) 1pt  t(14;20) 1pt  gain/amp 1q (1pt) | Composite Score ≥2 | 3 strata;  HRMM: 31% | Median:  28.6m//54m | Easy to apply;  Derived from non-trial population for derivation and a large proportion in intermediate risk, |
| Individualized risk in MM (IRMMa)^13^, (n=1938), 2024 | ISS stage, age, LDH, treatment | Various high-risk events  (including:deletions 17p/1p, gain 1q, CNVs, mutational signatures) | Individualized | Individualized | NA | Comprehensive molecular profile and incorporates treatment data;  Difficult to incoporate in routine practice;  Relatively short follow-up |
| Amp: amplification; β2M: beta-2 microglobulin; CNV: copy number variants; HRD: high-risk disease; IFM: Intergroupe Francophone du Myélome; IMWG: International Myeloma Working Group ISS: international staging system; pt=point; LDH: lactate dehydrogenase; m= months; NA: not applicable; PFS: progression-free survival; OS: overall survival; ULN: upper limit of normal; WES: whole exome sequencing | | | | | | |
